# Functional neurological disorders in childhood and adolescence: Epidemiology and phenomenology of an emerging diagnostic and clinical challenge

**DOI:** 10.1192/j.eurpsy.2021.664

**Published:** 2021-08-13

**Authors:** V. Baglioni, S. Cesario, F. Gigliotti, S. Galosi, C. Di Maggio, M. Ferrara, V. Leuzzi, F. Di Santo

**Affiliations:** 1 Department Of Human Neuroscience, Section Of Child And Adolescent Neuropsychiatry, Sapienza University of Rome, rome, Italy; 2 Department Of Human Neuroscience, Section Of Child And Adolescent Neuropsychiatry, Sapienza University of Rome, Rome, Italy

**Keywords:** Functional Motor Disorders, Psychogenic non-epileptic seizures, Functional Neurological Disorder, Psychogenic Disorder

## Abstract

**Introduction:**

Literature on childhood Functional Neurological Disorders (FNDs) is spare. Clinical presentations are vaguely characterized and often misdiagnosed in younger ages. Their main neurological features enrol: Psychogenic non-epileptic seizures (PNES), Functional movement disorders (FMDs), sensory alterations, cephalgia and feeding problems.

**Objectives:**

The study was aimed to better characterize the childhood population of FND, because of they represent an emerging challenge for clinicians, giving its higher presentation in the younger age and the difficulties of an early and differential diagnosis as well as an effective management.

**Methods:**

Our study retrospectively examined the characteristics of 82 FNDs children and adolescents (8 to 16 y.o.; 13 males; 29 females) referred as neurological inpatients of an urban academic neuropsychiatric department, from 2014 to 2019. Three main clinical aspects were analysed: type and pattern of symptoms manifestations (DSM-5 criteria); Life Events; family functioning.

**Results:**

FND accounted for 2% of 5-years consultations of neurological inpatients (M: F=1:2). The clinical presentation was characterized in 70% by pattern of co-expressed neurological symptoms: FMDs (9.5%); PNES (12%); dizziness/lipothymia (12%); paraesthesia/anaesthesia (16%). Generalized pain was associated in 38% of the reported patterns while cephalgia in 44%. Sleep disorders were reported in 40%. Previous psychiatric diagnoses were uncommon (2 out 82). Antecedent stressors were identified in 97% of patients for personal illness history and in the 93% for chronic illness in the family anamnesis. Family problems were in 25% of cases.

**Conclusions:**

Our data contributes to better characterize the childhood population of FND, describing clinical patterns of presentation, highlighting putative antecedent stressors and risk factors

